# Metformin for the management and prevention of antipsychotic-induced weight gain

**DOI:** 10.3892/mi.2026.312

**Published:** 2026-03-30

**Authors:** Sara Shreen, Mazher Ali, Syeda Lubna Eram, Mahreen Baig, Mohammed Abdul Nayeem, Waheed Fatima Asna Omer, Minhajzafar Nasirabadi, Syed Altaf Hussain

**Affiliations:** 1 Department of Pharmacy Practice, Deccan School of Pharmacy, Hyderabad, Telangana 500001, India; 2 Department of Psychiatry, Neelima Institute of Medical Sciences, Hyderabad, Telangana 500088, India; 3 Department of Psychiatry, Owaisi Hospital and Research Centre, Hyderabad, Telangana 500058, India; 4 Department of Pediatrics, Owaisi Hospital and Research Centre, Hyderabad, Telangana 500058, India

**Keywords:** antipsychotic-associated weight gain, metformin, second-generation antipsychotics, schizophrenia, metabolic adverse effects

## Abstract

Psychiatric disorders are commonly treated with antipsychotic medications; however, their use is frequently complicated by antipsychotic-associated weight gain (AAWG), which contributes to reduced treatment adherence and an increased cardiometabolic risk. Metformin, an insulin-sensitizing agent widely used in the management of diabetes, has emerged as a potential adjunctive therapy for mitigating AAWG, although real-world clinical data remain limited. The present prospective observational study evaluated the effectiveness of adjunctive metformin therapy in the management of AAWG in a routine psychiatric care setting. The present study was conducted over a period of 6 months and included 100 adult patients receiving antipsychotic treatment. Participants were allocated into two groups based on routine clinical practice: A test group receiving metformin in addition to antipsychotic therapy and lifestyle modifications (n=50), and a control group receiving antipsychotic therapy with lifestyle modifications alone (n=50). Changes in body weight and body mass index (BMI) were assessed as primary outcomes. The study population predominantly comprised younger adults aged 18-29 years, with a higher proportion of female patients. Olanzapine was the most frequently prescribed antipsychotic, and schizophrenia was the most common psychiatric diagnosis. The test group demonstrated a reduction in mean body weight and BMI over the study period, whereas the control group exhibited an increase in both parameters. Female patients reported a greater adherence to metformin therapy; however, adherence was assessed using self-reports and pill counts. These findings suggest that adjunctive metformin therapy may be associated with improvements in weight-related outcomes among patients receiving antipsychotics. Given the observational and non-randomized design, the results should be interpreted with caution, and further randomized controlled studies are warranted to confirm the long-term metabolic benefits.

## Introduction

Severe psychiatric disorders, including schizophrenia, bipolar disorder and related psychotic illnesses, are among the leading causes of long-term disability worldwide. Although antipsychotic medications remain indispensable for symptom control, relapse prevention and functional recovery, their long-term use is frequently complicated by adverse physical health outcomes. Among these, antipsychotic-associated weight gain (AAWG) represents one of the most common, distressing and clinically consequential adverse effects, contributing substantially to excess cardiometabolic morbidity and premature mortality in this population (1-3).

Individuals with severe mental illness experience a markedly reduced life expectancy compared with the general population, with cardiovascular disease accounting for a large proportion of excess deaths (1,2). Weight gain following the initiation of antipsychotics is a major modifiable contributor to this disparity. Clinically significant weight gain, often defined as a ≥7% increase from baseline body weight, has been reported with almost all antipsychotic agents, although the magnitude and trajectory of weight change vary across drugs and individuals (3-5). Notably, weight gain frequently emerges early in the course of treatment, progresses rapidly during the first months, and may persist long term, even when psychiatric symptoms are well-controlled (6,7).

Second-generation antipsychotics (SGAs), particularly olanzapine and clozapine, are consistently associated with the highest risk of AAWG and metabolic disturbances, including insulin resistance, dyslipidemia and impaired glucose tolerance (5,6). These adverse effects are not merely cosmetic; they are strongly associated with non-adherence to treatment, a reduced quality of life, stigma, and the early discontinuation of otherwise effective antipsychotic therapy (8,9). Consequently, AAWG represents a critical challenge at the intersection of psychiatric stability and physical health, underscoring the need for effective and acceptable management strategies.

**Figure 1:**
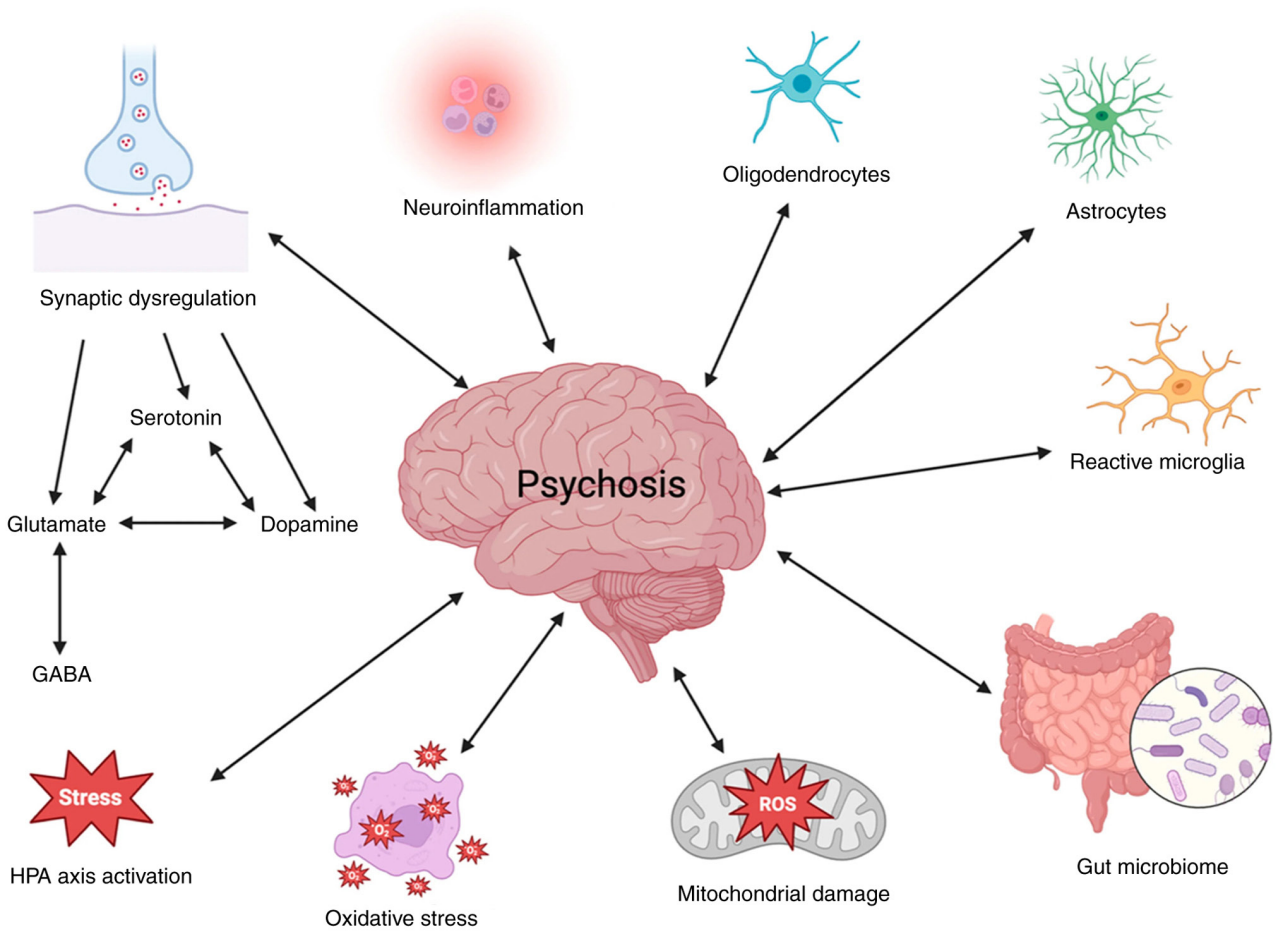
Potential interacting factors in the etiology of psychosis. The image was taken from a previous study (CC BY license (22).

The mechanisms underlying AAWG are multifactorial and involve the dysregulation of appetite control, satiety signaling, energy expenditure and peripheral metabolism. Antipsychotic medications interact with central neurotransmitter systems involved in energy homeostasis, including serotonergic, histaminergic, muscarinic and dopaminergic pathways, as well as peripheral metabolic processes that influence insulin sensitivity and lipid handling (10-12). The broader neurobiological framework of psychosis, including neurotransmission dysregulation, neuroinflammation, oxidative stress and mitochondrial dysfunction, is illustrated in Fig. 1. Although these mechanisms are central to psychotic disorders, only selected pathways are directly implicated in antipsychotic-associated weight gain (13).

From a clinical perspective, the trajectory of AAWG is particularly relevant. Longitudinal studies have demonstrated that the greatest proportion of weight gain occurs within the first 6-12 months of antipsychotic exposure, after which weight gain often plateaus at a higher set point (6,14). This pattern suggests that early intervention is crucial to prevent excessive long-term weight accumulation. Once a new weight set point is established, reversal becomes considerably more difficult, even with intensive lifestyle or pharmacological interventions (15). This understanding has critical implications for the timing and selection of preventive strategies.

Lifestyle modifications, including dietary counseling, increased physical activity and behavioral interventions, are is widely recommended as first-line management for AAWG in clinical guidelines (16-18). However, the real-world effectiveness of lifestyle interventions in patients with severe mental illness is often limited. Cognitive impairment, negative symptoms, sedation, social disadvantage and illness-related functional limitations can significantly reduce engagement and adherence (19,20). Moreover, structured lifestyle programs are resource-intensive and not consistently available, particularly in low- and middle-income countries. As a result, reliance on lifestyle modifications alone frequently fails to prevent clinically meaningful weight gain in routine psychiatric practice.

These limitations have prompted growing interest in adjunctive pharmacological strategies to mitigate AAWG. Among available options, metformin has emerged as the most extensively studied and consistently supported agent. Metformin is a well-established insulin-sensitizing drug with decades of use in the management of type 2 diabetes mellitus. Its mechanisms of action extend beyond glucose lowering and include effects on appetite regulation, gut hormone secretion, insulin sensitivity, and energy balance (12,21). Of note, metformin does not cause hypoglycemia when used as monotherapy and has a favorable safety profile when appropriately prescribed and monitored.

Evidence from randomized controlled trials and meta-analyses over the past two decades has demonstrated that metformin can attenuate AAWG and improve metabolic parameters in both diabetic and non-diabetic populations (22-25). More recent analyses have refined this understanding by highlighting the importance of timing. Meta-analyses and systematic reviews published within the past 5 years have indicated that metformin is particularly effective when initiated early in the course of antipsychotic treatment or in antipsychotic-naïve patients, where it can prevent further weight gain rather than attempting to reverse established obesity (24-27). This preventive effect is clinically meaningful, as even modest reductions in weight trajectory can translate into substantial long-term cardiometabolic benefit.

Perspectives in high-impact psychiatric literature, including Schizophrenia Bulletin, have emphasized the need to re-evaluate traditional stepwise algorithms that reserve metformin as a last-line option only after lifestyle modifications and antipsychotic switching have failed (18). Such approaches may inadvertently delay an effective intervention until the window for maximal benefit has passed. Instead, emerging evidence supports a more proactive and individualized approach, integrating metformin earlier in patients at a high risk of AAWG, particularly those prescribed high-risk antipsychotics or those experiencing rapid early weight gain.

Despite this growing evidence base, the real-world implementation of metformin for AAWG remains inconsistent. Clinician concerns regarding off-label use, perceived safety risks, pill burden, and uncertainty about long-term benefits continue to limit its uptake in psychiatric settings (28). In addition, the majority of available data are derived from controlled trials conducted in high-income countries, and there remains a relative paucity of prospective observational data reflecting routine clinical practice in diverse healthcare systems (13,19-21). This gap is particularly relevant in low- and middle-income countries, where resource constraints, limited access to structured lifestyle programs, and high reliance on metabolically adverse antipsychotics may amplify the burden of AAWG.

Furthermore, patient-centered considerations are increasingly recognized as essential in the management of AAWG. Qualitative research indicates that weight gain is among the most distressing side effects of antipsychotic treatment from the patient perspective, often perceived as more troubling than some psychiatric symptoms themselves (29). Patients frequently report a desire for early, effective interventions that acknowledge both physical and mental health priorities. Pharmacological strategies, such as metformin, which may reduce hunger and stabilize weight, are often viewed as acceptable and empowering when appropriately explained and monitored.

Against this background, there is a clear need for prospective, real-world studies evaluating the effectiveness of adjunctive metformin therapy for AAWG in routine psychiatric care. Such studies can complement randomized controlled trials by capturing broader patient populations, reflecting typical prescribing practices, and addressing pragmatic outcomes such as adherence and tolerability. Notably, observational data can help inform context-specific clinical decision-making, particularly in healthcare systems where guideline recommendations may not fully account for local constraints and patient preferences.

Therefore, the present prospective observational study was undertaken to evaluate the effectiveness of adjunctive metformin therapy, in combination with lifestyle modifications, in mitigating antipsychotic-associated weight gain in a real-world psychiatric population. By focusing on clinically meaningful outcomes such as changes in body weight and body mass index, and by situating the findings within contemporary evidence and evolving clinical perspectives, this study aims to contribute to the growing body of literature supporting earlier and more individualized management of AAWG.

## Patients and methods

### Study population

The present study was a 6-month, prospective observational, non-randomized study conducted at the Department of Psychiatry at Owaisi Hospital and Research Centre, Hyderabad, India, which provides both inpatient and outpatient psychiatric services. The primary objective was to assess the effectiveness of adjunctive metformin therapy in the management and prevention of AAWG. Clinical data were collected from the medical records of patients, including case sheets and treatment charts. Patient recruitment and data collection were conducted between June, 2024 and November, 2024. Eligible participants were adults aged 18-65 years, of either sex, who were receiving antipsychotic medications. The median age of the study population was 27 years. Metformin was prescribed at doses of 500, 850 mg, or 1 g according to routine clinical judgment. Participants were allocated into two groups based on usual prescribing practices: A test group receiving metformin in addition to antipsychotic therapy and lifestyle modifications, and a control group receiving antipsychotic therapy with lifestyle modifications alone. The baseline demographic and clinicopathological characteristics of the study population are summarized in Table I.

### Lifestyle modifications

Lifestyle modifications were provided to the participants in both study groups as part of routine clinical care. These consisted of standardized verbal counseling delivered by the treating psychiatrist or clinical pharmacist during outpatient visits or inpatient rounds. Dietary advice included recommendations for a balanced diet, reduction of calorie-dense and high-fat foods, limitation of sugar-sweetened beverages, and the encouragement of regular meal timing, in accordance with established guidelines for the management of antipsychotic-associated weight gain and cardiometabolic risk (6-8).

Physical activity advice focused on moderate-intensity activities, such as brisk walking for ~30 min per day on at least 3-5 days per week, tailored to the physical capacity and psychiatric status of the patient, consistent with international recommendations for individuals with severe mental illness (9,11). Educational counseling emphasized the association between antipsychotic therapy, weight gain and an increased cardiometabolic risk, as recommended by contemporary clinical guidelines (7,11).

No structured, supervised, or protocol-driven dietary or exercise program was implemented. The intensity and frequency of lifestyle interventions were not standardized, and formal adherence assessment was not performed. Compliance with lifestyle recommendations was assessed based on patient self-report during routine follow-up visits, a commonly used approach in real-world observational psychiatric studies (12,13).

### Statistical analysis

Statistical analyses were performed using SPSS version 25 software (IBM Corp.). Continuous variables are presented as the mean ± standard deviation, and categorical variables are summarized as frequencies and percentages. Within-group comparisons of body weight and body mass index (BMI) before and after the intervention were conducted using paired t-tests. Between-group comparisons of mean changes in weight and BMI were performed using independent samples t-tests. The comparison of BMI was analyzed using a two-way mixed ANOVA with time (baseline vs. follow-up) with the Bonferroni correction as the within-subject factor and study group as the between-subject factor. For between-group analyses, mean differences in change scores (follow-up minus baseline) were calculated, and corresponding 95% confidence intervals (CIs) were derived based on the standard error of the mean difference using the independent samples t-test model. The CIs were computed as: mean difference ± (t x standard error of the difference), where the t-value was obtained from the appropriate degrees of freedom for each comparison. All tests were two-tailed, and a P-value of <0.05 was considered to indicate a statistically significant difference.

## Results

### Baseline characteristics

The majority of the study participants were young adults aged 18-29 years (39%), while only 2% were aged >60 years. Female patients constituted a higher proportion of the study population (64%) compared with males. Olanzapine was the most frequently prescribed antipsychotic (34%), followed by risperidone (24%) and amisulpride (16%). Clonazepam (12%) was prescribed as an adjunctive medication and was not classified as an antipsychotic. Cariprazine (6%), quetiapine (4%), and aripiprazole (4%) were less commonly used. Schizophrenia was the most prevalent psychiatric diagnosis (43%), followed by bipolar disorder (17%) and anxiety disorders (14%). Other diagnoses included major depressive disorder (13%), psychosis not otherwise specified (4%), obsessive-compulsive disorder (3%), post-traumatic disorder (3%), absence seizures (2%), and alcohol dependence (1%) (Table I).

### Weight change analysis

An exploratory comparison indicated that patients with ongoing treatment with antipsychotics exhibited numerically lower weight gain than the treatment-naïve patients; however, this difference was not statistically significant (mean difference, 6 kg; P=0.47; 95% CI, -2.3 to 1.09) (Table II).

Between-group analysis demonstrated a statistically significant difference in weight change. Participants in group 1 (metformin plus lifestyle modifications) experienced a mean weight reduction of ~4 kg compared with group 2 (lifestyle modifications alone) (P<0.001; 95% CI, -7.5 to -2.4) (Table II).

Adverse drug reactions exhibited no significant association with weight change. Patients reporting adverse reactions had a mean weight increase of 0.263 kg compared with those without adverse reactions (P=0.833; 95% CI, -2.1 to 2.7). Sex-based analysis revealed no statistically significant difference in weight change between female and male patients (P=0.387; 95% CI, -0.88 to 2.27) (Table II).

### Comparison of body weight

**Figure 2:**
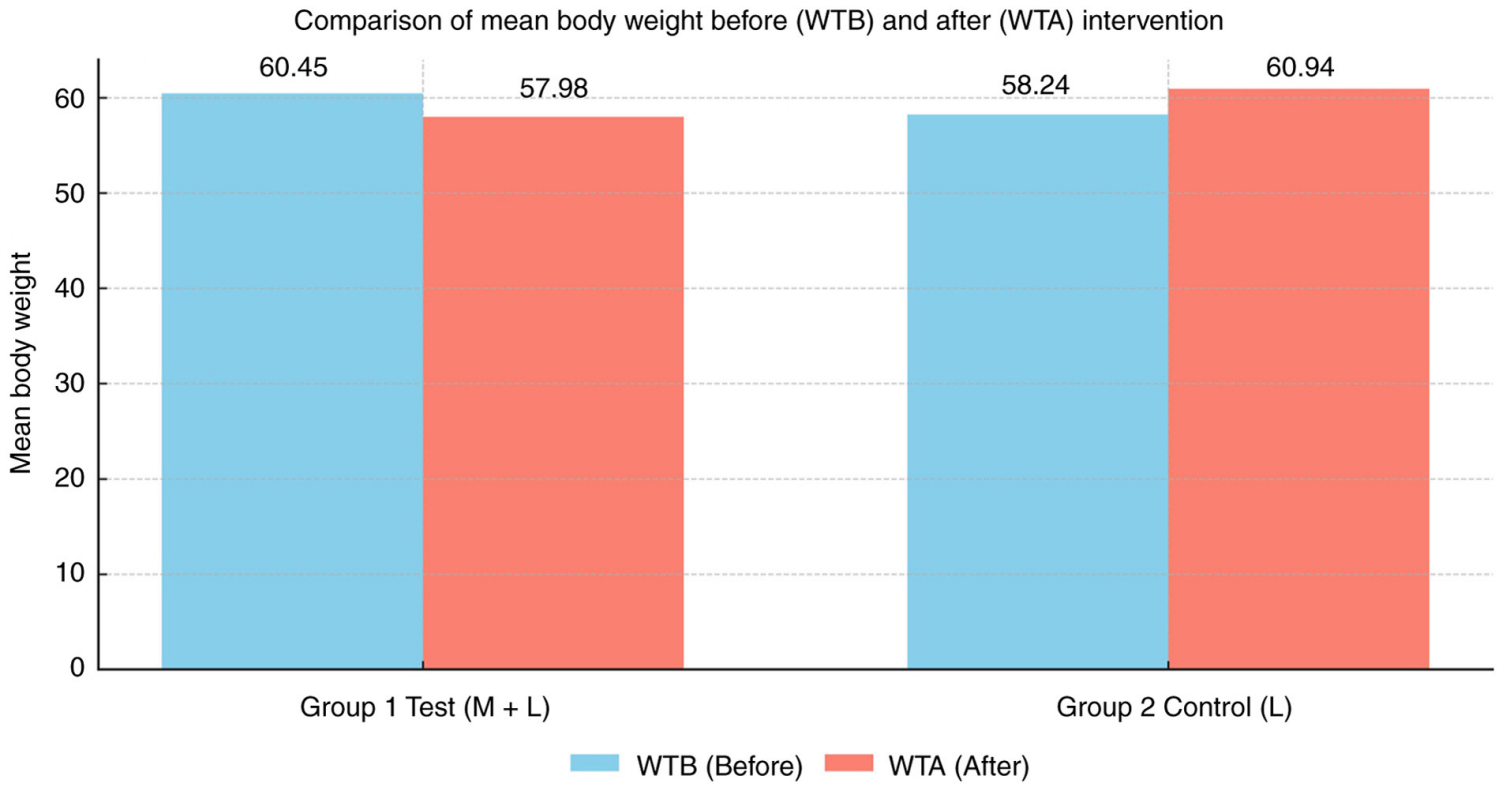
Comparison of mean body weight before (WTB) and after (WTA) the intervention in the test group (metformin + lifestyle modifications) and the control group (lifestyle modifications alone). The test group demonstrated a reduction in mean body weight following the intervention, whereas the control group exhibited an increase in mean body weight over the study period. Values are expressed as mean body weight (kg).

Within-group comparisons revealed that in group 1 (metformin plus lifestyle modifications), the mean body weight decreased from 60.45 kg at baseline to 57.98 kg following the intervention, indicating a net weight reduction. By contrast, group 2 (lifestyle modifications alone) demonstrated an increase in mean body weight from 58.24 to 60.94 kg over the study period. These findings indicate a beneficial effect of adjunctive metformin therapy in preventing antipsychotic-associated weight gain (Table III and Fig. 2).

### Comparison of BMI

BMI was assessed at baseline and after 6 months of follow-up in both study groups (Table IV). In group 1 (metformin plus lifestyle modifications), the mean BMI decreased from 23.6±3.4 kg/m^2^ at baseline to 22.6±3.3 kg/m^2^ at follow-up, representing a mean reduction of approximately 1.0 kg/m^2^, which was statistically significant (P<0.001).

The comparison of BMI was analyzed using a two-way mixed ANOVA with time (baseline vs. follow-up) as the within-subject factor and study group as the between-subject factor. The analysis demonstrated a significant main effect of time (P<0.001) and a significant group x time interaction (P<0.001), indicating that BMI changes over time differed between the two groups. Post-hoc comparisons with the Bonferroni correction revealed that the metformin group experienced a significant reduction in BMI from 23.6±3.4 to 22.6±3.3 kg/m^2^ (P<0.001). By contrast, the control group exhibited a significant increase in BMI from 22.9±3.2 to 24.0±3.5 kg/m^2^ (P<0.001). Between-group comparisons at follow-up also showed a significant difference favoring the metformin group (P=0.002) (Table IV).

## Discussion

In the present prospective observational study conducted in a real-world psychiatric setting, adjunctive metformin therapy was associated with a significant reduction in body weight and BMI among patients receiving antipsychotic treatment. Participants in the metformin group experienced a mean weight reduction of ~4 kg compared with those receiving lifestyle modification alone, supporting the effectiveness of metformin as an adjunctive strategy for managing AAWG. The study population reflects routine clinical practice, with a predominance of second-generation antipsychotics, particularly olanzapine and risperidone, agents well recognized for their high metabolic liability. Benzodiazepines, including clonazepam, were prescribed as concomitant medications and were not classified as antipsychotic agents for analytical purposes.

AAWG remains a major clinical challenge in long-term psychiatric care, contributing to increased cardiometabolic risk, reduced quality of life, and poor treatment adherence (14,15). Recent large-scale observational studies and meta-analyses have reaffirmed that SGAs such as olanzapine and clozapine are associated with the greatest magnitude of weight gain, particularly during the early phases of treatment (16,17). Contemporary evidence emphasizes that early and proactive intervention is critical, as weight gain trajectories established within the first 6-12 months are often sustained over time and difficult to reverse (18).

The findings of the present study are consistent with those of previous randomized controlled trials and meta-analyses published between 2021 and 2025, which demonstrate that adjunctive metformin significantly attenuates AAWG and improves metabolic parameters in patients receiving antipsychotic therapy (12,19-21). An updated meta-analysis by Zheng *et al* reported a clinically meaningful reduction in body weight and BMI with metformin compared with placebo, even among non-diabetic patients (21). Similarly, the study by Seifarth *et al* (20) confirmed moderate-quality evidence supporting metformin for reducing antipsychotic-related weight gain and insulin resistance. Other analyses have further highlighted that metformin is particularly effective when initiated early or in patients at high metabolic risk, reinforcing its role as both a preventive and therapeutic agent (21).

In the present study, longitudinal follow-up demonstrated a clear divergence in weight trajectories between groups. Patients receiving metformin exhibited a reduction in mean body weight from 60.45 to 57.98 kg, whereas those managed with lifestyle modifications alone experienced an increase from 58.24 to 60.94 kg. Corresponding changes in BMI mirrored these trends. These findings are consistent with previous randomized controlled trials and meta-analyses demonstrating significant reductions in body weight and BMI with adjunctive metformin in patients receiving antipsychotic therapy (3,21,24,27). Notably, these real-world data complement evidence from controlled trials by demonstrating effectiveness under routine prescribing conditions.

Mechanistically, metformin exerts its beneficial effects on weight regulation through multiple pathways, including improved insulin sensitivity, the suppression of hepatic gluconeogenesis, modulation of gut microbiota, and the enhancement of anorexigenic gut hormones such as glucagon-like peptide-1 and peptide YY (24,25). Recent translational studies suggest that these mechanisms may counteract antipsychotic-induced disruptions in appetite signaling and peripheral metabolism, thereby stabilizing weight trajectories in vulnerable patients (26). The observed benefits of metformin in non-diabetic populations further support its broader applicability in psychiatric practice (27).

An exploratory analysis in the present study suggested that patients with ongoing exposure to antipsychotics exhibited numerically lower weight gain compared with treatment-naïve individuals; however, this difference did not reach statistical significance and should be interpreted cautiously. Similar findings have been reported in recent cohort studies, where early metabolic changes were highly variable and influenced by baseline risk factors, antipsychotic type, and duration of exposure (28). These observations underscore the complexity of weight dynamics in psychiatric populations and highlight the need for individualized risk stratification.

Sex-based analysis revealed a higher adherence to metformin therapy among female patients, although no statistically significant difference in weight change between sexes was observed. Recent literature suggests that sex may influence medication adherence and health-seeking behaviors, with women often demonstrating greater engagement in weight-related interventions (29,30). However, adherence in the present study was assessed using self-report and pill counts rather than validated adherence instruments, limiting the strength of this conclusion. Nonetheless, these findings highlight the importance of considering psychosocial and behavioral factors when implementing metabolic interventions in psychiatric care.

Overall, the results of the present study add to the growing body of evidence supporting metformin as a safe and effective adjunctive intervention for mitigating AAWG, particularly when combined with lifestyle modification. Recent expert commentaries and consensus statements have increasingly advocated for earlier integration of metformin in high-risk patients, rather than reserving its use as a last-line option after significant weight gain has occurred (21,31). The present findings provide further real-world support for this evolving clinical approach.

Several limitations of the present study should be acknowledged. The single-center design may limit generalizability, and the 6-month follow-up period restricts assessment of long-term metabolic outcomes. The observational, non-randomized and non-blinded nature of the study introduces potential selection bias and residual confounding. Metformin dosing was guided by routine clinical practice, resulting in dose heterogeneity (500 mg to 1 g), which may have influenced treatment response. Lifestyle modifications were delivered as non-standardized counseling without objective adherence monitoring, limiting the ability to isolate the independent effects of metformin. Adherence assessment relied on patient self-report and pill counts rather than validated instruments, which may reduce precision, particularly in gender-based comparisons.

Despite these limitations, the present study provides valuable real-world evidence from a routine psychiatric setting. The findings should be interpreted with appropriate caution, and causal inferences cannot be definitively established. Future well-designed randomized controlled trials with longer follow-up are warranted to confirm these results, explore gender-specific adherence patterns, and evaluate the long-term cardiometabolic benefits of adjunctive metformin therapy.

In conclusion, the present prospective observational study demonstrated that adjunctive metformin therapy, when combined with lifestyle modifications, was associated with a statistically significant reduction in mean body weight and BMI among patients receiving antipsychotic treatment. Patients in the metformin group experienced a decrease in mean body weight from 60.45 to 57.98 kg, whereas those managed with lifestyle modifications alone showed an increase from 58.24 to 60.94 kg over the study period. These findings support the potential role of metformin in mitigating antipsychotic-associated weight gain in routine clinical practice. Although higher self-reported adherence to metformin therapy was observed among female patients, adherence was not assessed using validated instruments, and no statistically significant sex-based differences in weight outcomes were identified. Given the observational design, the findings should be interpreted with caution, and further well-designed randomized controlled trials are warranted to confirm these results and to evaluate long-term metabolic outcomes.

## Figures and Tables

**Table I:** Baseline demographic and clinicopathological characteristics of the study population.

Variable	No. of participants
Age (years), median (range)	27 (18-65)
Age group 18-29	39
Age group 30-39	-
Age group 40-49	-
Age group 50-59	-
≥60 years	2
Sex (female)	64
Sex (male)	36
Primary psychiatric diagnosis	
Schizophrenia	43
Bipolar disorder	17
Anxiety disorders	14
Major depressive disorder	13
Others	13
Antipsychotic prescribed	
Olanzapine	34
Risperidone	24
Amisulpride	16
Others	26

**Table II:** Comparison of mean change in body weight across clinical subgroups.

Variable	Mean weight change (kg)	Standard deviation	P-value	95% confidence interval
Treatment status				
Ongoing antipsychotic use	-0.62	2.31	0.47	-2.34 to 1.09
Treatment-naïve	Reference	-	-	-
Study group				
Test group (metformin + lifestyle modifications)	-4.99	1.29	<0.001	-7.53 to -2.47
Control group (lifestyle modifications only)	Reference	-	-	-
Adverse drug reactions (ADRs)				
Present	+0.26	1.25	0.83	-2.18 to 2.71
Absent	Reference	-	-	-
Sex				
Female	+1.69	0.80	0.39	-0.88 to 2.27
Male	Reference	-	-	-

Data were analysed using an independent samples t-test. Only the comparison between study groups exhibited a statistically significant difference in mean weight change.

**Table III:** Comparison of mean body weight before and after the intervention.

Parameter	Group 1: Test (metformin + lifestyle modifications)	Group 2: Control (lifestyle modifications only)
Weight before intervention (kg)	60.45±9.1	58.24±8.9
Weight after intervention (kg)	57.98±8.8	60.94±9.3
Mean change (kg)	-2.47	+2.70
Within-group P-value	<0.001	<0.001

Data were analysed using the paired t-test (within-group comparisons) and the independent samples t-test (between-group comparisons). Values are presented as the mean ± standard deviation. Weight change was calculated as weight after intervention minus weight before intervention.

**Table IV:** Comparison of BMI before and after the intervention.

A, BMI in the groups before and after the intervention		
Parameter	Group 1: Test (metformin + lifestyle modifications)	Group 2: Control (lifestyle modifications only)
BMI before intervention (kg/m^2^)	23.6±3.4	22.9±3.2
BMI after intervention (kg/m^2^)	22.6±3.3	24.0±3.5
B, Results of two-way mixed ANOVA		
Effect	F value	P-value
Time effect	F (1,98)=21.4	P<0.001
Group effect	F (1,98)=9.6	P=0.003
Time x group interaction	F (1,98)=28.7	P<0.001
C, Post-hoc Bonferroni comparisons		
Comparison	Mean difference	P-value
Test group: Baseline vs. follow-up	-1.0 kg/m²	P<0.001
Control group: Baseline vs. follow-up	+1.1 kg/m²	P<0.001
Between groups at follow-up	-1.4 kg/m²	P=0.002

BMI, body mass index.

## Data Availability

The data generated in the present study may be requested from the corresponding author.
